# Integrating Food Preference Profiling, Behavior Change Strategies, and Machine Learning for Cardiovascular Disease Prevention in a Personalized Nutrition Digital Health Intervention: Conceptual Pipeline Development and Proof-of-Principle Study

**DOI:** 10.2196/75106

**Published:** 2025-08-13

**Authors:** Hana Fitria Navratilova, Anthony David Whetton, Nophar Geifman

**Affiliations:** 1 School of Biosciences, Faculty of Health and Medical Sciences, University of Surrey, Leggett Building, Manor Park, Daphne Jackson Road, Guildford, GU2 7WG, United Kingdom; 2 Department of Community Nutrition, Faculty of Human Ecology, IPB University, Bogor, Indonesia; 3 Veterinary Health Innovation Engine, School of Veterinary Medicine, University of Surrey, Guildford, United Kingdom; 4 School of Health Sciences, Faculty of Health and Medical Sciences, University of Surrey, Kate Granger Building, 30 Priestley Road, Surrey Research Park, Guildford, GU2 7YH, United Kingdom, 44 1483686700

**Keywords:** behaviour change, diet, food preference, UK Biobank, risk prediction

## Abstract

**Background:**

Personalized dietary advice needs to consider the individual’s health risks as well as specific food preferences, offering healthier options aligned with personal tastes.

**Objective:**

This study aimed to develop a digital health intervention (DHI) that provides personalized nutrition recommendations based on individual food preference profiles (FPP), using data from the UK Biobank.

**Methods:**

Data from 61,229 UK Biobank participants were used to develop a conceptual pipeline for a DHIs. The pipeline included three steps: (1) developing a simplified food preference profiling tool, (2) creating a cardiovascular disease (CVD) prediction model using the subsequent profiles, and (3) selecting intervention features. The CVD prediction model was created using 3 different predictor sets (Framingham set, diet set, and FPP set) across 4 machine learning models: logistic regression, linear discriminant analysis, random forest, and support vector machine. Intervention functions were designed using the Behavior Change Wheel, and behavior change techniques were selected for the DHI features.

**Results:**

The feature selection process identified 14 food items out of 140 that effectively classify FPPs. The food preference profile prediction set, which did not include blood measurements or detailed nutrient intake, demonstrated comparable accuracy (across the 4 models: 0.721-0.725) to the Framingham set (0.724-0.727) and diet set (0.722-0.725). Linear discriminant analysis was chosen as the best-performing model. Four key features of the DHI were identified: food source and portion information, recipes, a dietary recommendation system, and community exchange platforms. The FPP and CVD risk prediction model serve as inputs for the dietary recommendation system. Two levels of personalized nutrition advice were proposed: level 1—based on food portion intake and FPP; and level 2—based on nutrient intake, FPP, and CVD risk probability.

**Conclusions:**

This study presents proof of principle for a conceptual pipeline for a DHI that empowers users to make informed dietary choices and reduce CVD risk by catering to person-specific needs and preferences. By making healthy eating more accessible and sustainable, the DHI has the potential to significantly impact public health outcomes.

## Introduction

Cardiovascular diseases (CVDs), including conditions such as coronary heart disease, cerebrovascular disease, and peripheral arterial disease, are among the leading causes of death worldwide, accounting for an estimated 17.9 million deaths annually (32% of global deaths) [1]. Poor diet quality, as well as other lifestyle factors, are significant modifiable risk factors for CVDs [2,3]. These risks can be mitigated through lifestyle changes, such as adopting a healthier diet, increasing physical activity, and reducing tobacco and alcohol use [4,5]. Embracing these habits is crucial for both preventing and managing CVD, as recommended in guidelines [6,7]. Notably, a healthy diet ranks sixth for myocardial infarction and fourth for stroke out of the 10 risk factors in the population attributable risk [3], suggesting that improving dietary habits can have a substantial effect on reducing the risk of these conditions.

Personalized nutrition has the potential to improve health, enhance well-being, and lower the risk of diet-related diseases more effectively than traditional dietary recommendations. These often fail to account for personal food preferences, leading to suboptimal adherence and limited effectiveness of prescribed restrictive diets [8]. One key issue in dietary intervention is adherence to a healthy diet. Using algorithms to tailor dietary advice based on clinical markers and individual diet preferences can effectively scale personalized nutrition recommendations. However, there is still limited evidence that tailoring a diet solely based on individual biological differences improves cardiometabolic health. It also remains uncertain whether this approach yields better outcomes compared with an optimal generic diet [9].

Using data from the UK Biobank, 3 participant profiles based on food preferences have been identified using machine learning [10]. These food preference participant profiles, which were found to be associated with differing disease risks, exhibit group-distinctive blood-based metabolomic and proteomic characteristics, which can potentially be used to tailor dietary recommendations more effectively. This approach would consider individual preferences to address disease risk and potential barriers to dietary changes. By understanding food preferences, personalized interventions can be created that are more likely to be adhered to, ultimately helping to reduce disease risk and improve overall health outcomes. This study provides a conceptual framework for a digital health intervention (DHI), which incorporates interventions by linking behavior understanding to proven techniques, offering personalized nutrition recommendations, with a particular focus on the role of dietary fats in CVD prevention as an exemplar.

## Methods

### Datasets

Data from the UK Biobank (accessed under application number: 83988), inclusive of computer-based questionnaire, health-related records, and biomarker measurements on over 500,000 older adult participants recruited in the United Kingdom between 2006 and 2010, were used. In this study, only participants who completed the Food Preference Questionnaire (FPQ) and at least 3 repeated 24-hour recall dietary data (n=61,229) were selected and included in the analysis. Participants with CVD were identified based on *ICD-10* (*International Classification of Diseases, Tenth Revision*) codes recorded as either the primary or secondary diagnosis. All diseases under Chapter IX (Diseases of the Circulatory System) were considered.

### Food Preference Profile Classifier

Food preference clusters were previously identified using latent profile analysis applied to 140 food item liking scores, using the mclust package in R (version 6.1; R Foundation for Statistical Computing) [10]. Briefly, the mclust package automates parameter estimation for the best-fit model using the expectation-maximization algorithm, which iteratively maximizes the likelihood of the data given the model parameters. The analysis tested for any number of profiles between 2 and 9 and identified the ellipsoidal, equal volume, and equal shape model with 3 resulting profiles as the best fit, based on the Bayesian information criterion and the additional requirement that each profile contain at least 10% of participants (Multimedia Appendix 1). Next, participants were exclusively assigned to 1 of the 3 identified profiles (named “Health-conscious,” “Omnivore,” and “Sweet-tooth”) based on their highest posterior probability. These profiles were further investigated for associations with different conditions, clinical outcomes, and biomarkers [10]. The profile assignments, along with the data used to identify them, were used in the next step. To reduce the number of questions needed to accurately assign participants to their profiles (from 140), a decision tree model was used. At each node of the tree, the algorithm selected the question and threshold that best separates the participants into distinct food preference profiles (FPPs). To ensure the robustness and accuracy of the decision tree, feature importance analysis was conducted using a random forest (RF), least absolute shrinkage and selection operator (LASSO) regression, and Shapley Additive Explanations (SHAP) values.

### CVD Prediction Model

The prediction goal was measured per participant, focusing on the risk of developing CVD using a prognostic model. Four standard machine learning models were considered to predict CVD: logistic regression, linear discriminant analysis, RF, and support vector machine. All models were implemented using the caret package in R. Data imputation for all models was conducted using the k-nearest neighbors algorithm, resulting in 5 imputed datasets. The data were split into 70% for training and 30% for testing.

Three different training sets were created based on distinct sets of predictors. The first predictor set, referred to as the Framingham set, included traditional Framingham risk factors [11]: continuous variables in this set were age, sex, systolic blood pressure, diastolic blood pressure, total cholesterol, high-density lipoprotein, BMI, and waist circumference (WC). Categorical variables included smoking status, treatment for hypertension, and history of hypertension. The second predictor set, referred to as the diet set, was based on nutrient intake. Continuous variables in this set were average daily intake of energy, protein, fat, carbohydrate, alcohol, and fiber, along with age, sex, BMI, and WC. Categorical variables included smoking status, treatment for hypertension, and history of hypertension. The third predictor set, termed the FPP set, combined FPPs with nonblood measurement Framingham risk factors. Continuous variables in this set were age, sex, BMI, and WC. Categorical variables included smoking status, treatment for hypertension, history of hypertension, and FPP. The occurrence of CVD is treated as a binary outcome (present or absent).

To avoid overfitting, we evaluated the prediction accuracy of all models using 10-fold stratified cross-validation. Model prediction performance was measured based on accuracy, area under the curve, and area under the precision-recall curve. The best-performing model was subsequently integrated into the recommendation system to predict the risk of developing CVD for each participant in the testing dataset, based on their FPP. Due to the unavailability of suitable external datasets, this study does not include external validation.

### Selection of DHI Key Features Based on Theoretically Driven Behavioral Intervention

**Figure 1.**
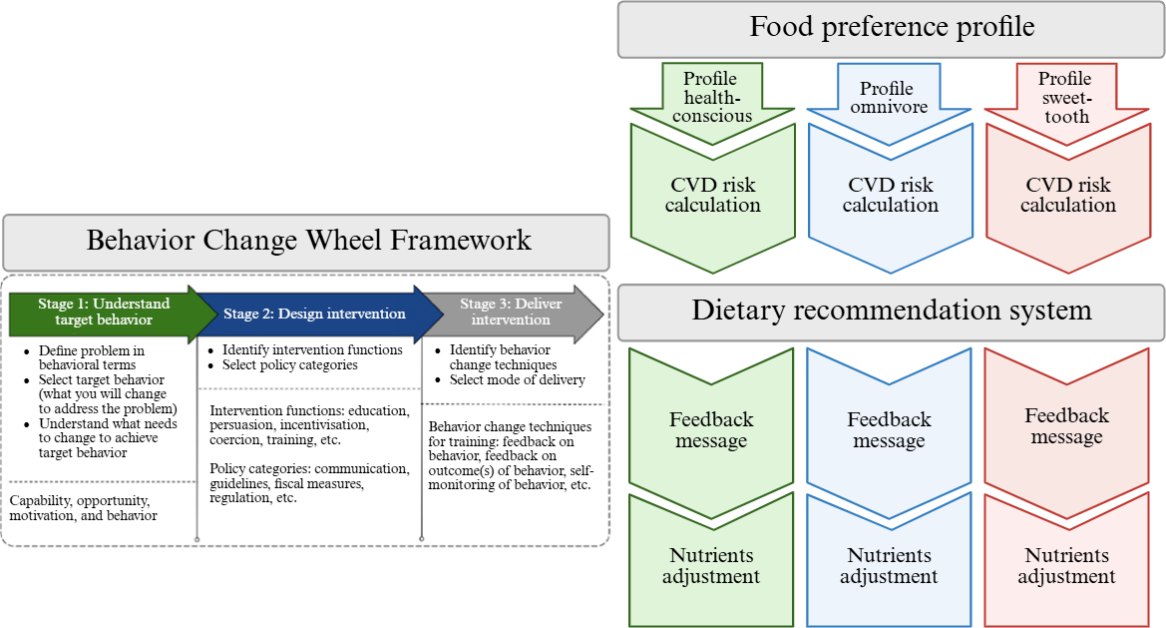
Stages in the development of the digital health intervention. CVD: cardiovascular disease.

Target behaviors appropriate for CVD prevention were selected based on the capability, opportunity, motivation–behavior (COM-B) model, which provides a comprehensive framework for understanding behavior change [12]. This was further analyzed using the Theoretical Domains Framework (TDF) to identify specific barriers and facilitators related to dietary changes, particularly those involving fats, through a literature review [13-18]. Key features for the DHI were determined by incorporating selected behavior change techniques (BCTs) relevant to the identified barriers. To ensure user-friendliness, design guidelines were developed to address barriers to prolonged use of digital health platforms [19]. Stages for developing the DHI are shown in Figure 1. The first step involves classifying users into one of the three FPPs: health-conscious (HC), omnivore (O), or sweet-tooth (ST). Next, a CVD prediction model calculates the risk for each profile. These profiles and risk assessments serve as inputs for the recommendation system. Dietary intervention, a key feature of the DHI, is based on BCTs that were systematically developed using the Behavior Change Wheel (BCW) framework. 

### Development of the Dietary Recommendation System

Following the selection of key features for the DHI, the next step involved developing the necessary algorithms to build a dietary recommendation system. Two levels of personalized nutrition advice are proposed: level 1—advice based on food portion intake and FPP data; level 2—advice based on nutrient intake, FPP, and CVD risk probability. The first algorithm provides level 1 advice by linking food intake data to feedback messages tailored to user profiles (health-conscious, omnivore, or sweet-tooth). The second algorithm delivers level 2 advice by connecting nutrient intake data to nutrient adjustment recommendations based on the user’s CVD risk and FPP.

As the aim of the DHI is related to healthy fat intake, the focus of food item entry for level 1 was planned as animal-based protein food, including oily fish, nonoily fish, processed meat, poultry, and red meat. The Eatwell Guide, specifically designed for the UK population, was used as a basis for setting the reference portion of protein foods. Feedback messages and provided information are based on the British Nutrition Foundation balanced diet portion size list [20].

Six nutrients were selected for level 2 advice: energy, protein, saturated fatty acids, trans-fatty acids, omega-3, and omega-6 intake, as these nutrients are associated with CVD. To set up nutrient reference intakes tailored to each profile (nutrient change required, upper limit, and lower limit), we assessed intercluster differences in the association between each nutrient and CVD. The best-performing model’s coefficients were also used to estimate the amount of nutrient change required to reduce the log (odds) of CVD:


(1) $$∆N_{ij}=∆R{{(\beta }_{ij})}^{-1}$$

where $∆N_{ij}$ is the required change for each nutrient (j) for profile (i), ∆R is the target decrease in CVD risk for each participant, and $\beta_{ij}$ is the coefficient for nutrient (j) from profile (i).

To assess the impact of nutrient intake levels on CVD onset across various FPPs, the Kaplan-Meier Estimate was used. This approach determined the optimal upper (${max\_val}_{ij}$) and lower (${min\_val}_{ij})$) limits of each nutrient intake, for each profile, based on the best survival curves. To predict time-to-event based on intake levels, intake data were divided into 4 quintiles. The observations were right-censored at 68 years to minimize survivor bias, and participants diagnosed before the first 24-hour dietary recall assessment date were removed to ensure that diet was not influenced by a previous diagnosis of any CVD. In addition, we developed a set of loss functions to penalize deviations from the proposed nutrient changes for each FPP. Thus, the recommended adjusted nutrient intake ($I_{ij}’$) follows this:

$I_{ij}’$ = $I_{ij}$ + $∆N_{ij}$if P > 0.41 & $I_{ij}$ < ${min\_val}_{ij}$, or

$I_{ij}’=$$I_{ij}$ - $∆N_{ij}$ if P > 0.41 & $I_{ij}$ > ${max\_val}_{ij}$ (2)

where $I_{ij}$ is the intake for each nutrient (j) for profile (i), P is the predicted probability of CVD for each participant, ${min\_val}_{ij}$ and ${max\_val}_{ij}$ is the minimum and maximum optimal values for each nutrient (j) from profile (i). Dietary decision trees were then manually developed to provide level 1 and level 2 dietary feedback advice.

### Ethical Considerations

The UK Biobank has ethical approval from the North West Multicenter Research Ethics Committee (REC reference number: 16/NW/0274) under its Research Tissue Bank framework. This approval permits researchers to conduct health-related research using UK Biobank data without requiring additional ethical clearance for each project. Written informed consent was obtained for all UK Biobank study participants. The UK Biobank’s consent process includes provisions for secondary analysis without the need for additional consent. The study data were pseudonymized by the UK Biobank, ensuring that the sample IDs are unique and random for each project, thereby maintaining the anonymity and deidentification of the data. Consequently, no further ethical approval was necessary for this secondary data analysis. In addition, this study received institutional approval from the Research Integrity and Governance Office at the University of Surrey (FHMS 22‐23 273 EGA).

## Results

### FPP Classifier

FPPs were previously derived from latent profile analysis, defining 3 distinct profiles. The first profile, termed health-conscious, shows a higher preference for fruits and vegetables, with a lower preference for meat, sweets, and fatty foods. The second profile, termed omnivore, includes preferences across almost all food groups, with a higher preference for meat and fish, while avoiding strongly flavored foods. The third profile, termed sweet-tooth, had a lower preference for most foods except for sweetened beverages and sweet foods [10].

**Figure 2.**
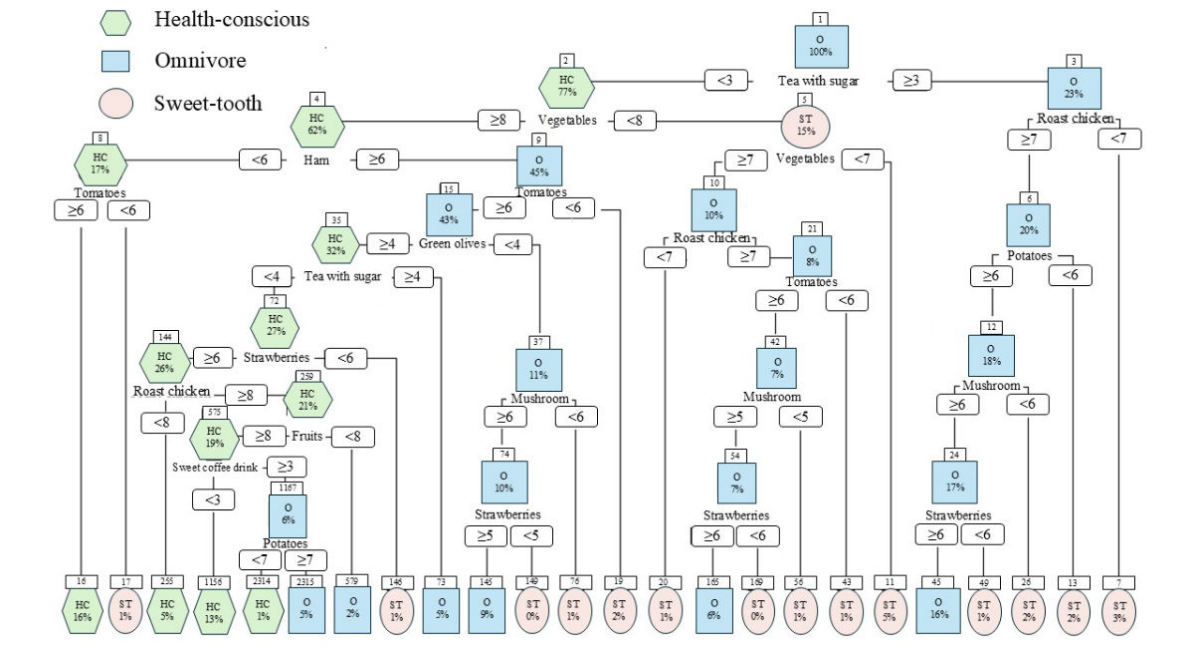
Decision tree for simplified food preference profile classifier.

To identify the most important features for reducing the number of questions respondents needed to answer, as well as the liking score threshold to classify them into profiles, a decision tree analysis was performed. The analysis identified 14 food items with sufficient classification power for FPPs: tea with sugar, vegetables, roast chicken, ham, tomatoes, strawberries, green olives, banana, sweet coffee drinks, coffee with sugar, fruit, mushroom, spinach, and potatoes. Each box in the decision tree represents a node. The top number in each box is the node number. The label inside the box includes two rows: the predicted class for that node (first row) and the distribution of samples across the tree (second row). The percentage values shown are the proportion of observations in that node relative to the total number of observations in the dataset. The decision tree model indicated that “tea with sugar” was the most significant predictor for omnivores. Node 1, representing this predictor, had a threshold score of <3. The initial split at Node 1 resulted in 2 subsequent nodes. Node 2, corresponding to a liking score for “tea with sugar”<3, was further split based on “vegetable ≥8.” This node predominantly predicted the health-conscious group. Conversely, node 3, corresponding to a score for “tea with sugar” ≥3, was split based on “roast chicken≥7.” This node predominantly predicted for the omnivore. Participants were classified as part of the sweet-tooth group if their liking score for roast chicken was less than 7 (Figure 2).

The food items selected by the decision tree were validated using RF, LASSO regression, and SHAP values (Multimedia Appendices 2-4). The RF algorithm identified the following features as the most important for classifying FPPs: fresh tomatoes, tea with sugar, coffee with sugar, mushrooms, vegetables, strawberries, ham, sausage meat, bacon, and spinach. Further analysis using a LASSO regression approach highlighted the key dietary features that differentiate each group. The health-conscious group was characterized by negative associations with tea with sugar, while the omnivore group showed positive associations with roast chicken. The sweet-tooth group, on the other hand, had negative associations with tomatoes and strawberries. These results were also consistent with SHAP values.

The classification model’s performance was evaluated using sensitivity, specificity, and *F*1 metrics across the three classes. The specificity values were 0.84 for health-conscious, 0.75 for omnivore, and 0.93 for sweet-tooth, while the sensitivity values were 0.75 for health-conscious, 0.73 for omnivore, and 0.58 for sweet-tooth. The high specificity values indicate the model results in few false positives. Furthermore, the *F*1-scores for the classes health-conscious, omnivore, and sweet-tooth, which were 0.71, 0.69, and 0.66, respectively, indicate a good level of performance for the prediction model across the different classes. Nevertheless, classification could be improved, particularly in enhancing the prediction accuracy for the class sweet-tooth.

### CVD Risk Prediction Model

Given the differences in CVD prevalence across different food preference groups, we calculate the risk probability for each person by considering their specific food preference group. At the group level, the health-conscious group had the lowest odds ratio (OR) of 0.74 (95% CI 0.71‐0.77), indicating a lower risk of CVD. In contrast, the omnivore and sweet-tooth groups had similar and higher odds ratio (OR 1.16, 95% CI 1.11‐1.20 and OR 1.17, 95% CI 1.12‐1.22, respectively), indicating a higher risk of CVD. These differences necessitated tailored prediction models to provide accurate and personalized recommendations for each group.

To determine the most accurate method for predicting CVD risk, we evaluated 3 different predictor sets across 4 machine learning models. Among the 3 predictor sets, the Framingham set demonstrated the highest accuracy, ranging from 0.724 to 0.727 across all machine learning models. Although the FPP set showed slightly lower accuracy (0.721-0.725) compared with the diet set (0.722-0.725), its performance remains commendable. This indicates that the FPP can effectively predict CVD risk, even without the need for blood measurements and detailed nutrient intake as predictors, potentially offering a more practical and scalable approach to health risk prediction. The comparable predictive performance of the FPP model suggests that it captures partially orthogonal information—providing distinct behavioral insights that are not fully represented in traditional clinical predictors such as those in the Framingham model. This complementary value is particularly relevant in contexts where clinical data may be unavailable or difficult to obtain. Overall, all 3 predictor sets exhibited similar accuracy, with the linear discriminant analysis model emerging as the best-performing model. Notably, both the simplified and full versions of the FPP predictor set (Multimedia Appendix 5) demonstrate comparable predictive accuracy, underscoring its robustness and potential utility in the DHI (Table 1).

**Table 1.** Cardiovascular disease risk prediction performance of 4 models using 3 different predictor sets.

Model	Accuracy	Harrell C statistic	Area under the curve	Area under the precision-recall curve
Logistic regression				
Framingham^a^	0.726	0.678	0.759	0.679
Diet^b^	0.725	0.676	0.756	0.677
Food preference profile^c^	0.724	0.675	0.755	0.676
Linear discrimination analysis				
Framingham	0.727	0.677	0.758	0.678
Diet	0.725	0.674	0.755	0.676
Food preference profile	0.725	0.674	0.755	0.676
Random forest				
Framingham	0.726	0.677	0.756	0.679
Diet	0.725	0.671	0.748	0.676
Food preference profile	0.723	0.668	0.745	0.675
Support vector machine				
Framingham	0.724	0.674	0.674	0.583
Diet	0.722	0.671	0.671	0.581
Food preference profile	0.721	0.670	0.670	0.580

a Framingham predictor set: age, sex, systolic blood pressure, diastolic blood pressure, total cholesterol, high-density lipoprotein, BMI, waist circumference, smoking status, treatment for hypertension, and history of hypertension.

b Diet predictor set: average daily intake of energy, protein, fat, carbohydrate, alcohol, and fiber, age, sex, BMI, waist circumference, smoking status, treatment for hypertension, and history of hypertension.

c Food preference profile predictor set: food preference profile, age, sex, BMI, waist circumference, smoking status, treatment for hypertension, and history of hypertension.

### DHI Key Features

To design an effective intervention for behavior change, the first step is to clearly define the behavior that needs to be changed. We identified modification of fat intake to reduce CVD risk in older adults as a suitable behavior for a DHI. Next, 2 target behaviors were selected: choosing healthy fats and cooking methods. Several barriers that might hinder the achievement of these target behavioral approaches were identified, including negative attitudes toward increasing total fat intake and the taste of key foods, lack of knowledge about food types and proportions, limited cooking skills, resistance to changing eating habits, increased costs, and the need for supervision and encouragement. These barriers are categorized and described using the COM-B framework and the TDF (Table 2).

**Table 2.** Intervention component matrix.

COM-B^a^ component	TDF^b^	Barrier	Intervention function	Facilitation	BCTs^c^	Application	Feature
Physical capability	Physical skill	Limited cooking skills	Education	Offering recipes to enhance culinary skills and confidence in preparing healthy meals	Action planning	Creating action plans for meal preparation	Recipe
Psychological capability	Knowledge	Lack of knowledge about food types and proportions	Education	Providing information and resources to improve understanding of healthy fats and appropriate portion sizes	Information about health consequences and credible source	Providing detailed information on the health benefits of healthy fats and using credible sources to enhance trust and understanding	Food source and portion information
Social opportunity	Social influences	Need for supervision and encouragement	Enablement	Nudges and reminders	Feedback on behavior, self-monitoring of behavior, or social support (unspecified)	Providing regular feedback, tools for self-monitoring, and social support to maintain motivation and adherence	Dietary feedback
Physical opportunity	Environmental context and resources	Increased costs	Enablement	Providing tips for budget-friendly healthy eating and information on affordable sources of healthy fats	Goal setting (behavior) and action planning	Setting realistic goals for budget-friendly healthy eating and planning actions to achieve them	Recipe
Reflective motivation	Beliefs about capabilities	Resistance to changing eating habits	Persuasion	Crafting persuasive messages and campaigns to promote the benefits of healthy fats	Prompts or cues, social support (unspecified)	Using reminders and social support systems to encourage gradual changes in eating habits	Dietary feedback
Reflective motivation	Beliefs about consequences	Negative attitudes toward increasing total fat intake and the taste of key foods	Persuasion	Using positive messaging and testimonials to change perceptions and highlight the benefits and palatability of healthy fats	Feedback on outcomes of the behavior, credible source	Sharing positive outcomes and testimonials from credible sources to change perceptions	Community exchange platforms
Automatic motivation	Reinforcement	Negative attitudes toward increasing total fat intake and the taste of key foods	Persuasion	Using positive messaging and testimonials to change perceptions and highlight the benefits and palatability of healthy fats	Feedback on outcomes of the behavior and credible source	Sharing positive outcomes and testimonials from credible sources to change perceptions	Community exchange platforms

a COM-B: capability, opportunity, motivation–behavior.

b TDF: Theoretical Domains Framework.

c BCT: behavior change technique.

Following the COM-B analysis, the intervention functions that met the APEASE (Acceptability, Practicability, Effectiveness, Affordability, Side-effects, and Equity) criteria included education, enablement, and persuasion. Several potential policy categories align with these intervention functions, such as communications and marketing, guidelines, regulation, legislation, service provision, fiscal measures, and environmental and social planning. However, in the context of the DHI, the policy categories that meet the APEASE criteria are communications and marketing, guidelines, and service provision. Subsequently, BCTs were selected to address the sources of behavior, TDF domains, and intervention functions. These BCTs include information about health consequences, feedback on behavior, feedback on the outcome of behavior, prompts or cues, self-monitoring of behavior, credible sources, social support, goal setting (behavior), adding objects to the environment, and action planning. Finally, 4 key features of the DHI were identified: food source and portion information, recipes, dietary feedback, and community exchange platforms.

### Artificial Intelligence–Based Dietary Recommendation System

**Figure 3.**
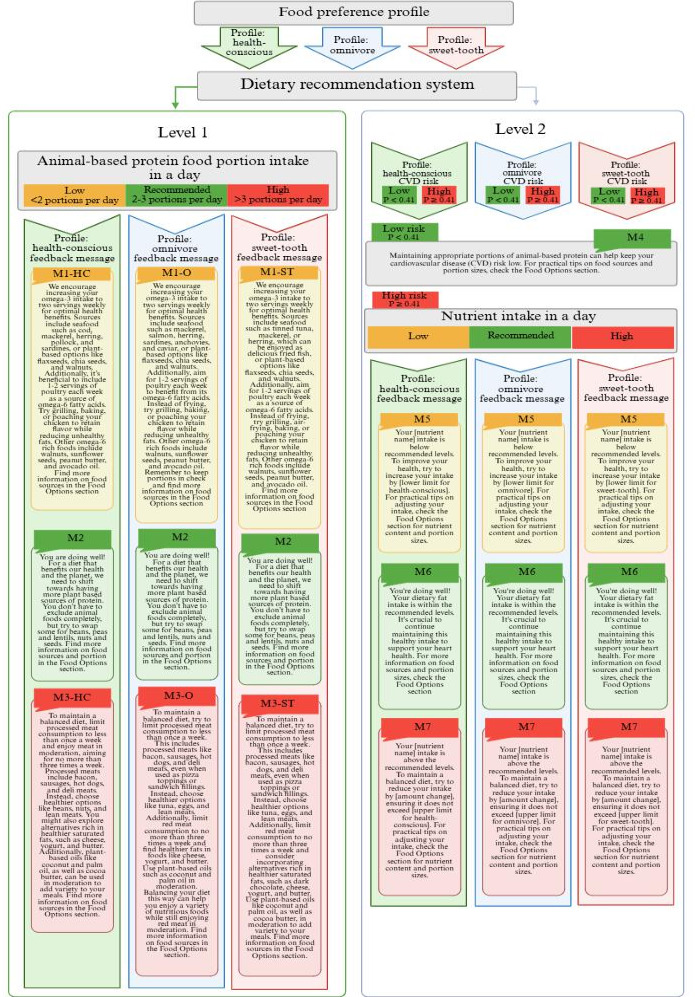
Workflow diagram and corresponding feedback messages. HC: health-conscious; O: omnivore; ST: sweet-tooth.

To provide personalized nutrition guidance that addresses individual dietary preferences and CVD risk, the dietary recommendation system is divided into 2 levels: personalized messages tailored to each user’s profile, focusing on portion sizes and food options, and calculations of dietary changes required to reduce CVD risk (Figure 3). Users can choose to receive either level 1 advice (portion suggestions message from M1-M3), level 2 advice (nutrient adjustments message from M4-M7), or both. The process is designed so that accessing level 2 advice does not require completion of level 1 advice. This design addresses identified barriers to digital interventions, such as the dislike of calorie counting and the time-consuming nature of food entry. By offering 2 levels of advice, the feedback system eliminates these barriers while providing customizable and tailored options to meet individual needs and goals.

Given the distinct food preferences of the 3 profiles, personalized messages regarding food sources and portions can be adjusted accordingly. When a portion is detected as lower than the reference, message M1 encourages increased consumption, focusing on lean meat (poultry) and fish, with specific food options aligned with each profile’s preferences (M1-HC, M1-O, and M1-ST). Conversely, when a portion is detected as higher than the reference, message M3 encourages a reduction in red meat and processed meat consumption, with profile-specific food options (M3-HC, M3-O, and M3-ST). The algorithm does not account for vegetarian or vegan users; thus, these users will receive the M1 recommendation. However, this message also includes suggestions for plant-based food options. Level 2 advice begins with calculating the user's CVD risk. Users with low CVD risk receive M4 messages. In contrast, those with high risk receive feedback based on their nutrient intake levels: M5 is provided for low-level intake, M6 for recommended intake, and M7 for high intake. 

The testing dataset for the disease prediction model split the low and high risk at a threshold of 0.41 for all 3 profiles (Figure 3), which provided the best accuracy. The nutrient intake modifications required to reduce the risk of CVD by 1%, along with the optimal intake ranges for each profile, are presented in Table 3. The objective of a 1% risk reduction is to decrease the probability of being classified as having the disease by reducing the likelihood of falling on the “disease present” side of the 0.41 threshold. The CVD risk prediction model estimates were then used to determine the direction and magnitude of nutritional adjustments. All groups were required to decrease their target nutrient intake to reduce the risk of CVD. The most notable change is for the omnivore group, which needs to reduce its energy intake by 623 kJ/day (148 kcal/day) to lower the risk of CVD by 1%. In comparison, the health-conscious group requires a reduction of 307 kJ/day (73 kcal/day), and the sweet-tooth group needs a reduction of 325 kJ/day (77 kcal/day). The calculated reductions are designed to be effective while ensuring that nutrient intake does not fall below recommended lower limits.

**Table 3.** Conversion from compositional coefficients of cardiovascular diseases (CVDs) for each profile to the intake required to reduce the risk of CVDs by 1% for level 2 advice.

Nutrient	Health-conscious (required change (lower-upper limit))	Omnivore (required change (lower-upper limit))	Sweet-tooth (required change (lower-upper limit))
Energy (kJ/d)	−307 (8136-9360)	−623 (7486-9965)	−325 (7252-9773)
Protein (g/d)	−4.72 (64.74-87.85)	−3.19 (70.67-92.96)	−3.48 (66.61-90.47)
SFA (g/d)^a^	−3.07 (13.41-21.97)	−2.79 (14.81-23.71)	−3.06 (14.35-23.37)
TFA (g/d)^b^	−0.30 (0-1.42)	−0.33 (0-1.54)	−0.37 (0-1.48)
Omega 3 (g/d)^c^	−0.31 (1.47-2.46)	−0.45 (1.49-2.42)	−0.39 (1.41-2.33)
Omega 6 (g/d)^d^	−0.45 (7.98-13.08)	−0.42 (8.32-13.14)	−0.42 (8.07-13.06)

a WHO recommends limiting saturated fatty acid (SFA) intake to less than 10% of total energy intake (~<22 grams/day for a 2000 kcal diet).

WHO recommends limiting trans-fatty acid (TFA) intake to less than 1% of total energy intake (~<2.2 grams/day for a 2000 kcal diet).

Although WHO does not specify a precise daily gram intake, a general recommendation is to consume 0.5%-2% of total energy from omega-3 fatty acids, which corresponds to approximately 1.1–1.6 grams/day for a 2000 kcal diet.

WHO suggests an intake of 2.5%-9% of total energy from omega-6 fatty acids, equivalent to roughly 12-17 grams/day for a 2000 kcal diet.

The upper and lower intake limits for each profile are based on the best quintiles from the Kaplan-Meier Estimate, defined as the quintiles with the earliest and latest onset of disease. Using these quintiles ensures that the dietary recommendations are grounded in empirical evidence, reflecting the most and least favorable outcomes observed in the population. The resulting nutrient thresholds largely align with World Health Organization (WHO) recommendations. No lower limit is set for trans-fatty acids due to the absence of a known safe consumption level. Consequently, the goal is to minimize or eliminate trans-fat intake.

## Discussion

### Principal Findings

Here we present the conceptual design, framework development, and initial implementation of a DHI aimed at empowering individuals to make informed dietary choices, using the reduction of CVD risk as an exemplar use case. Empowering individuals to direct their own lifestyle modifications is more cost-effective when such lifestyle factors play a role in disease progression. Although the target nutrients are limited, this design establishes a preliminary pipeline that incorporates behavior change interventions and food preferences. The 2 levels of advice—level 1 (portion suggestions) and level 2 (nutrient adjustments)—are intended to keep the DHI flexible and enable it to be customized. This approach seeks to increase adherence to a healthy diet by addressing barriers to dietary change and digital intervention while allowing users to choose the level of guidance that best suits their needs and preferences.

The 4 selected features serve the intervention functions of education, enablement, and persuasion. The first feature, food source and portion information, provides detailed insights into the health benefits of healthy fats, using credible sources to enhance trust and understanding. The Eatwell Guide is used to cater to the UK population, as it has been scientifically developed using modeling to balance different foods and drinks, ensuring the recommended nutrient intake [20]. This alignment prevents conflicting information when users seek advice from dietitians or health care professionals.

The second feature, recipes, assists in creating action plans for meal preparation, setting realistic goals for budget-friendly healthy eating, and planning actions to achieve them. Although such interventions have not been significantly associated with changes in cardiometabolic risk factors [21], this feature specifically aims to address barriers related to limited cooking skills and potential increased costs. By providing practical and affordable meal options, the expected outcome is an improvement in users’ attitudes toward cooking and their self-efficacy in preparing healthy meals.

The simplified FPP classification and CVD prediction model were built as part of the third feature, the dietary feedback system. The simplified classifier, which uses 14 food items out of the full 140 food items, demonstrates good accuracy. Our analysis using LASSO and SHAP values indicates that it is easier to identify features that differentiate the health-conscious group from the other two, as this group is associated with more distinctive dietary patterns. In contrast, the omnivore and sweet-tooth groups often share similar feature associations, making it more challenging for the model to differentiate between them. This overlap likely contributes to the lower recall for the sweet-tooth group. Nonetheless, the intervention remains focused on promoting behavior change to improve dietary habits, which is expected to be beneficial regardless of classification nuances. The primary goal is to encourage healthier eating patterns; thus, even with moderate sensitivity, the model can identify a significant portion of the target population. In addition, we have included both lower and upper limits for nutrient intake that align with general health dietary guidelines. This ensures that the dietary recommendations are balanced and promote overall health, making the intervention suitable for the sweet-tooth group despite the model’s moderate performance.

The Framingham prediction set on its own demonstrated an accuracy range from 0.71 to 0.72 [22,23], while dietary scores alone reported a discrimination performance ranging from 0.773 to 0.776 for CVD [24]. Remarkably, the FPP set achieved similar performance without requiring invasive blood-measurement data and detailed dietary data. The FPP set offers significant advantages, such as eliminating potential recall bias and reducing the time required for users to input their daily food intake, making it more user-friendly and practical.

Decision tree algorithms were developed to provide personalized feedback on intakes of 5 animal-based protein foods and 6 nutrients. This feedback system was designed to deliver relevant advice for adults at higher risk of CVD; hence the limited focus on specific foods and nutrients. The feedback system also aims to improve adherence to dietary advice by addressing individual food preferences. Previous studies found that personalized nutrition has been shown to enhance adherence to a healthier diet and consequently improve health outcomes [25,26].

Focusing dietary feedback on fatty acids instead of total fat helps avoid unintended consequences, such as increased consumption of refined carbohydrates and added sugars, and the avoidance of nutrient-dense foods rich in healthy unsaturated fats such as nuts, seeds, avocados, and vegetable oils. Consequently, the recommendation to increase portions of animal-based protein foods emphasizes fish and poultry, which contain healthier dietary fats, while reducing portions of red meat and processed meat. In addition, the recommendations include suggestions for plant-based protein sources, such as legumes, tofu, and quinoa, to ensure a balanced approach for individuals with varied dietary preferences. Our analysis reveals that the optimal range for each profile, determined by food preferences, aligns with general guidelines for fatty acid intake [27]. In addition, the advice covers not only food options but also cooking methods, such as avoiding fried foods to reduce trans-fat intake. The advice is formulated in accordance with established guidelines [28], thus ensuring that scientific information is effectively communicated and translated for public understanding.

### Comparison With Previous Work

The 2 levels of dietary advice are used here to improve adherence. A previous study by Horne et al [29] found that individuals with multiple genetic variants and genetically based nutrition recommendations were advised to focus on achieving 1 nutrition target of their choosing before moving on to another when they felt ready for further dietary changes. This approach led to greater adherence to the diet compared with the control group.

Furthermore, the focus on limited nutrients and food groups for intervention is supported by findings from the Food4Me Study, which emphasizes the importance of targeting selected top-priority food-based dietary goals for personalized dietary interventions [30]. The study suggests that individuals are more likely to adhere to dietary recommendations when they are simple and directly related to everyday food choices, rather than complex nutrient calculations. By prioritizing key food-based goals, personalized interventions can be tailored to address the most impactful dietary behaviors, thereby enhancing overall health outcomes and adherence.

### Limitations

One limitation of this study is the inability to provide a direct assessment of the application of the pipeline. Second, while the general definition of personalized nutrition should include both behavioral analysis and biological evidence (such as genetic differences) to predict responses to interventions [31], it is also important to consider that FPPs are associated with distinctive blood-based metabolic and proteomic profiles [10]. Notably, differences in leptin expression have been observed, demonstrating that each preference profile elicits a different metabolic response.

Finally, the UK Biobank cohort primarily consists of middle-aged adults from the United Kingdom, which may limit the ability to generalize our findings to other demographic groups and geographic regions. In addition, the model lacks external validation due to the lack of access to comparable cohorts. While the algorithm for food preference profiling and CVD prediction was derived specifically from the UK Biobank population, the overall conceptual pipeline can be implemented with different datasets from various populations. Furthermore, the system could be extended to include other dietary factors such as sodium, fiber, or added sugars, further enhancing its applicability and relevance across diverse dietary patterns and health outcomes.

### Future Directions

Future research will focus on testing the recommendation system to provide effective and personalized recommendations within a web-based tool. Improving the model’s accuracy will involve strategies such as class-specific threshold tuning, cost-sensitive learning, and enhanced feature engineering. The proposed user interface design includes a personalized dashboard that features 4 key DHI components: food source and portion information, recipes, dietary feedback, and community exchange platforms. During the sign-up process, users’ FPPs are determined, allowing personalized messages to be automatically tailored to each user’s profile. These messages focus on portion sizes, food options, and calculations of dietary changes required to reduce CVD risk. Dietary feedback messages are delivered upon input of recent food intake. The system will generate feedback messages using computer algorithms and a relational database based on the assigned FPP. To support adherence, users can track their progress through visual charts provided in the dashboard. Future deployment will require consideration of regulatory and ethical factors, including GDPR (General Data Protection Regulation) compliance for handling personal health data and potential medical device classification under UK and EU (European Union) regulations. As suitable independent datasets that encompass all the required input data become available, external validation can be performed. Candidate cohorts include the Netherlands Twin Register [32], the Avon Longitudinal Study of Parents and Children [33], and the All of Us Research Program [34], which offer rich phenotypic and genetic data suitable for replication.

### Conclusions

This study develops a conceptual pipeline for a dietary intervention tool using UK Biobank data, incorporating BCTs to provide personalized nutrition recommendations aimed at reducing dietary fats and preventing CVD. The CVD prediction model, based on food preference inputs, offers a noninvasive and simplified alternative for cardiovascular risk assessment. Two levels of advice are designed to enhance adherence to dietary recommendations by enabling users to choose the guidance that best aligns with their needs and preferences. This DHI conceptual pipeline has the potential to be implemented as an effective personalized nutrition tool.

## Supplementary material

10.2196/75106 Multimedia Appendix 1 Latent profile analysis of 140 food items reveals 3 distinct profiles.

10.2196/75106 Multimedia Appendix 2 Feature importance analysis using least absolute shrinkage and selection operator.

10.2196/75106 Multimedia Appendix 3 Feature importance analysis using SHAP (Shapley Additive Explanations) value.

10.2196/75106 Multimedia Appendix 4 Feature importance analysis using random forest.

10.2196/75106 Multimedia Appendix 5 Comparison of cardiovascular disease risk prediction performance using linear discriminant analysis between the full food preference profile model (140 food liking scores) and the simplified food preference profile model (14 food liking scores).

## Abbreviations

APEASE: Acceptability, Practicability, Effectiveness, Affordability, Side-effects, and Equity
BCT: behavior change technique
BCW: Behavior Change Wheel
COM-B: capability, opportunity, motivation – behavior
CVD: cardiovascular disease
DHI: digital health intervention
EU: European Union
FPP: food preference profile
FPQ: Food Preference Questionnaire
GDPR: General Data Protection Regulation
HC: health conscious
*ICD-10*: *International Statistical Classification of Diseases, Tenth Revision*
LASSO: least absolute shrinkage and selection operator
O: omnivore
OR: odds ratio
RF: random forest
SHAP: Shapley Additive Explanations
ST: sweet tooth
TDF: Theoretical Domain Framework
WC: waist circumference
WHO: World Health Organization
