# Supplementary material for: Integrating Food Preference Profiling, Behavior Change Strategies, and Machine Learning for Cardiovascular Disease Prevention in a Personalized Nutrition Digital Health Intervention: Conceptual Pipeline Development and Proof-of-Principle Study
Source: J Med Internet Res. 2025 Aug 13;27:e75106. doi: 10.2196/75106 (PMC12346185; doi:10.2196/75106)
Supplement: Multimedia Appendix 1 [file jmir-v27-e75106-s001.docx]

Supplementary data 1. Latent Profile Analysis of 140 Food Items Reveals Three Distinct Profiles

| Food items | X1 (Health-conscious) | X2 (Omnivore) | X3 (Sweet-tooth) |
| --- | --- | --- | --- |
| Bacon | -0.46 | 0.39 | 0.01 |
| BBQ grilled meat | -0.38 | 0.35 | -0.04 |
| Beef steak | -0.43 | 0.36 | 0.01 |
| Bolognese sauce | -0.21 | 0.34 | -0.21 |
| Chicken | -0.36 | 0.38 | -0.09 |
| Fried chicken | -0.46 | 0.34 | 0.06 |
| Ham | -0.48 | 0.41 | 0 |
| Lamb | -0.32 | 0.34 | -0.08 |
| Liver | -0.14 | 0.25 | -0.16 |
| Pork chops | -0.42 | 0.39 | -0.05 |
| Red meat | -0.46 | 0.38 | 0.01 |
| Roast chicken | -0.36 | 0.38 | -0.09 |
| Salami | -0.15 | 0.23 | -0.13 |
| Sausages | -0.47 | 0.37 | 0.03 |
| Burgers | -0.48 | 0.34 | 0.08 |
| Baked/steam fish | 0.1 | 0.22 | -0.38 |
| Cod | -0.09 | 0.3 | -0.29 |
| Fried fish | -0.39 | 0.34 | -0.01 |
| Haddock | 0 | 0.27 | -0.35 |
| Herring | 0.11 | 0.12 | -0.27 |
| Mackerel | 0.13 | 0.18 | -0.37 |
| Pollock | 0.11 | 0.07 | -0.21 |
| Prawns | 0.01 | 0.23 | -0.31 |
| Salmon | 0.07 | 0.26 | -0.4 |
| Sardines | 0.1 | 0.19 | -0.34 |
| Shellfish | 0.11 | 0.13 | -0.28 |
| Smoked fish | 0.06 | 0.21 | -0.33 |
| Tinned Tuna | -0.05 | 0.25 | -0.27 |
| Asparagus | 0.4 | 0.02 | -0.46 |
| Beetroot | 0.24 | 0.07 | -0.35 |
| Broad beans | 0.32 | 0.02 | -0.37 |
| Broccoli | 0.37 | 0.11 | -0.54 |
| Brussel sprout | 0.21 | 0.1 | -0.36 |
| Cabbage | 0.24 | 0.12 | -0.42 |
| Cauliflower | 0.25 | 0.14 | -0.45 |
| Cucumber | 0.22 | 0.12 | -0.39 |
| Lentil beans | 0.37 | 0.02 | -0.42 |
| Mushroom | 0.18 | 0.19 | -0.44 |
| Onion | 0.14 | 0.13 | -0.32 |
| Salad leaves | 0.37 | 0.08 | -0.49 |
| Spinach | 0.46 | -0.01 | -0.49 |
| Turnip | 0.15 | 0.08 | -0.27 |
| Vegetables | 0.4 | 0.1 | -0.55 |
| Strawberries | 0.15 | 0.27 | -0.49 |
| Orange juice | -0.27 | 0.28 | -0.08 |
| Oranges | 0.1 | 0.23 | -0.4 |
| Banana | -0.05 | 0.31 | -0.35 |
| Tomatoes | 0.29 | 0.25 | -0.64 |
| Fruit | 0.29 | 0.18 | -0.54 |
| Kiwi | 0.21 | 0.15 | -0.42 |
| Apple juice | -0.14 | 0.21 | -0.13 |
| Apple | 0.14 | 0.17 | -0.38 |
| Melon | 0.14 | 0.19 | -0.39 |
| Pears | 0.16 | 0.16 | -0.38 |
| Plums | 0.26 | 0.19 | -0.51 |
| Cherries | 0.29 | 0.13 | -0.48 |
| Grapefruit | 0.11 | 0.11 | -0.26 |
| Dried fruit | 0.15 | 0.14 | -0.34 |
| Lemons | 0.29 | 0.05 | -0.38 |
| Raw carrots | 0.29 | 0.06 | -0.38 |
| Skimmed milk | -0.03 | 0.08 | -0.08 |
| Whole grain cereals | 0.05 | 0.17 | -0.27 |
| Wholemeal bread | 0.11 | 0.17 | -0.34 |
| Cereal bar | -0.11 | 0.18 | -0.1 |
| Pasta | -0.01 | 0.22 | -0.27 |
| Porridge | 0.09 | 0.11 | -0.24 |
| Honey | -0.01 | 0.17 | -0.2 |
| Plain yogurt | 0.31 | 0.04 | -0.38 |
| Brown rice | 0.26 | 0.03 | -0.33 |
| Soy milk | 0.21 | -0.09 | -0.1 |
| Avocados | 0.44 | -0.02 | -0.44 |
| Blue cheese | 0.17 | 0.09 | -0.3 |
| Black olives | 0.41 | -0.03 | -0.4 |
| Capers | 0.34 | -0.05 | -0.3 |
| Gherkins | 0.24 | 0.05 | -0.32 |
| Globe artichoke | 0.39 | -0.13 | -0.26 |
| Green olives | 0.4 | -0.02 | -0.41 |
| Horseradish/wasabi | 0.21 | 0.03 | -0.27 |
| Bell pepper | 0.3 | -0.03 | -0.28 |
| Bitter foods | 0.2 | -0.02 | -0.2 |
| Black pepper | 0.24 | 0.04 | -0.3 |
| Burn spicy | 0.06 | 0 | -0.07 |
| Chilli pepper | 0.17 | 0.01 | -0.19 |
| Coriander | 0.33 | -0.03 | -0.31 |
| Curry | 0.09 | 0.1 | -0.22 |
| Garlic | 0.26 | 0.04 | -0.32 |
| Soy sauce | 0.07 | 0.13 | -0.24 |
| Spicy foods | 0.13 | 0.05 | -0.2 |
| Aniseed | 0.11 | 0.02 | -0.14 |
| Dark chocolate | 0.11 | 0.06 | -0.2 |
| Bitter ale | -0.09 | 0.11 | -0.05 |
| Lager | -0.13 | 0.15 | -0.05 |
| Red wine | 0.1 | 0.1 | -0.23 |
| Spirits | -0.05 | 0.14 | -0.12 |
| Whisky | -0.07 | 0.1 | -0.05 |
| White wine | 0.06 | 0.13 | -0.23 |
| Biscuits | -0.38 | 0.27 | 0.06 |
| Cake | -0.28 | 0.25 | -0.02 |
| Cake icing | -0.34 | 0.24 | 0.07 |
| Cheesecake | -0.29 | 0.28 | -0.04 |
| Cream | -0.26 | 0.28 | -0.07 |
| Croissant | -0.14 | 0.18 | -0.09 |
| Ice cream | -0.32 | 0.29 | -0.02 |
| Jam | -0.29 | 0.28 | -0.05 |
| Marzipan | -0.03 | 0.12 | -0.12 |
| Milk chocolate | -0.37 | 0.26 | 0.06 |
| Sweet foods | -0.35 | 0.24 | 0.07 |
| Chips | -0.34 | 0.26 | 0.03 |
| Pizza | -0.2 | 0.25 | -0.1 |
| Crisps | -0.23 | 0.2 | -0.01 |
| Potatoes | -0.17 | 0.22 | -0.09 |
| Dairy products | -0.2 | 0.3 | -0.17 |
| Hard cheese | -0.02 | 0.27 | -0.31 |
| Savoury biscuits | -0.09 | 0.21 | -0.17 |
| Soft cheese | 0.05 | 0.21 | -0.32 |
| Goat cheese | 0.27 | 0.02 | -0.31 |
| Mayonnaise | -0.07 | 0.22 | -0.19 |
| Salad dressing | -0.02 | 0.22 | -0.26 |
| Ketchup | -0.21 | 0.22 | -0.05 |
| Vinegar | -0.02 | 0.13 | -0.14 |
| Aubergine | 0.44 | -0.03 | -0.43 |
| Butternut squash | 0.41 | -0.04 | -0.39 |
| Salty foods | -0.11 | 0.11 | -0.01 |
| Salty pretzels | -0.01 | 0.08 | -0.09 |
| Add salt to food | -0.15 | 0.11 | 0.02 |
| Fatty foods | -0.29 | 0.18 | 0.09 |
| Sweet coffee drinks | -0.37 | 0.22 | 0.12 |
| Tea with sugar | -0.49 | 0.2 | 0.28 |
| Coffee with sugar | -0.51 | 0.25 | 0.23 |
| Coffee without sugar | 0.3 | -0.05 | -0.26 |
| Tea without sugar | 0.21 | 0.01 | -0.23 |
| EVO oil | 0.33 | 0.01 | -0.37 |
| Cornflakes | -0.43 | 0.28 | 0.11 |
| Diet fizzy drinks | -0.31 | 0.17 | 0.12 |
| Fizzy drinks | -0.41 | 0.23 | 0.15 |
| Butter on bread | -0.25 | 0.22 | -0.02 |
| White bread | -0.41 | 0.24 | 0.14 |
| Whole milk | -0.23 | 0.19 | 0 |
| White rice | -0.21 | 0.26 | -0.1 |
