# Supplementary material for: Integrating Food Preference Profiling, Behavior Change Strategies, and Machine Learning for Cardiovascular Disease Prevention in a Personalized Nutrition Digital Health Intervention: Conceptual Pipeline Development and Proof-of-Principle Study
Source: J Med Internet Res. 2025 Aug 13;27:e75106. doi: 10.2196/75106 (PMC12346185; doi:10.2196/75106)
Supplement: Multimedia Appendix 2 [file jmir-v27-e75106-s002.docx]

Supplementary data 2. Feature important analysis using LASSO

| Feature | Health-conscious | Omnivore | Sweet-tooth |
| --- | --- | --- | --- |
| Add salt to food | -1.27E-03 | 0.004317 |  |
| Aniseed |  | -0.01532 | 0.013439 |
| Apple | -3.22E-02 | 0.030931 |  |
| Apple juice | 5.60E-03 |  | -0.00871 |
| Asparagus | 1.06E-01 |  |  |
| Aubergine | 3.84E-02 |  | -0.01061 |
| Avocados | 9.77E-02 |  | -0.03292 |
| Bacon | -2.37E-02 | 0.040464 |  |
| Baked/steam fish | | -0.0203 | 0.005072 |
| Banana | -1.73E-02 | 0.317064 |  |
| BBQ grilled meat | | 0.001649 | -0.03166 |
| Beef steak | -4.31E-02 |  | 0.002998 |
| Beetroot | 5.54E-02 | -0.0303 |  |
| Bell pepper | 3.70E-04 |  | -0.01222 |
| Biscuits | -2.21E-02 |  | 0.009677 |
| Bitter ale | 6.68E-03 |  | -0.02007 |
| Bitter foods | -6.87E-03 |  | 0.013363 |
| Black olives | | -0.02818 | 0.019181 |
| Black pepper | 1.96E-02 | -0.03247 |  |
| Blue cheese | 2.11E-02 |  | -0.01756 |
| Bolognese sauce | | 0.077403 | -0.04652 |
| Broad beans | 4.14E-02 | -0.0088 |  |
| Broccoli | 1.73E-01 |  | -0.13842 |
| Brown rice | 9.17E-03 | -0.02746 |  |
| Brussel sprout | 3.12E-02 |  | -0.02847 |
| Burgers | -4.08E-02 |  | 0.007478 |
| Burn spicy |  | -0.01683 | 0.049428 |
| Butter on bread | -6.42E-02 |  | 0.015415 |
| Butternut squash | 3.03E-02 |  | -0.0102 |
| Cabbage | -6.42E-03 | 0.00979 |  |
| Cake | 1.37E-02 |  | -0.00938 |
| Cake icing | -2.83E-02 |  |  |
| Capers |  | -0.01275 |  |
| Cauliflower | 1.53E-03 |  | -0.09777 |
| Cereal bar | -8.52E-03 |  |  |
| Cheesecake | -1.03E-02 | 0.01375 |  |
| Cherries | 2.24E-01 |  | -0.04726 |
| Chicken | -8.62E-02 | 0.194692 |  |
| Chilli pepper | -4.92E-03 |  | 0.006202 |
| Chips |  | 0.002915 | -0.00937 |
| Cod | -9.37E-02 | 0.15181 |  |
| Coffee with sugar | -3.39E-01 | 0.006379 |  |
| Coffee without sugar | 1.72E-02 | -0.02044 |  |
| Coriander | 6.01E-03 | -0.0195 |  |
| Cornflakes | -3.96E-02 |  | 0.034366 |
| Cream | -3.02E-02 | 0.033369 |  |
| Crisps | -1.30E-02 |  | 0.019768 |
| Croissant |  | -0.00212 | 0.016485 |
| Cucumber | 2.39E-02 |  | -0.0149 |
| Curry | 1.68E-02 |  | -0.02604 |
| Dairy products | -3.27E-02 | 0.079533 |  |
| Dark chocolate | | -0.00701 | 0.006569 |
| Diet fizzy drinks | -4.56E-02 |  | 0.015702 |
| Dried fruit# | 9.07E-03 | -0.00595 |  |
| Eggs |  | -0.05085 | 0.048173 |
| EVO oil |  | -0.06013 |  |
| Fatty foods | 6.12E-03 |  | -0.02419 |
| Fizzy drinks | -1.44E-01 | 0.023489 |  |
| Fried chicken | -9.92E-03 |  | 0.024877 |
| Fried fish | -7.04E-02 | 0.001866 |  |
| Fruit | 3.03E-01 |  | -0.0956 |
| Garlic | 2.48E-02 | -0.03474 |  |
| Gherkins | 4.87E-03 |  | -0.00708 |
| Globe artichoke | 9.59E-03 |  | -0.04317 |
| Goat cheese | 6.21E-03 | -0.00786 |  |
| Grapefruit | 3.19E-03 | -0.00136 |  |
| Green olives | 3.09E-02 |  | -0.08189 |
| Haddock |  | 0.064388 | -0.02181 |
| Ham | -6.16E-02 | 0.026408 |  |
| Hard cheese | -1.71E-02 | 0.212208 |  |
| Herring |  | -0.02437 | 0.010558 |
| Honey | -6.98E-05 |  |  |
| Horseradish/wasabi | 1.41E-02 |  | -0.01181 |
| Ice cream | -6.09E-02 |  |  |
| Jam |  | 0.012894 | -0.00653 |
| Ketchup |  | 0.011651 | -0.00104 |
| Kiwi | -1.20E-02 |  | 0.001378 |
| Lager | 6.24E-03 |  | -0.01008 |
| Lamb | -8.05E-03 | 0.018212 |  |
| Lemons |  | -0.00305 | 0.029678 |
| Lentil beans | 1.04E-01 |  | -0.01158 |
| Liver |  | 0.013438 | -0.00701 |
| Mackerel | 3.03E-02 |  | -0.02335 |
| Marzipan | 1.39E-02 | -0.00388 |  |
| Mayonnaise | -1.30E-02 |  | 0.002873 |
| Melon |  | 0.057201 | -0.02161 |
| Milk chocolate | -4.59E-02 |  | 0.019957 |
| Mushroom |  | 0.031451 | -0.11982 |
| Onion |  |  | -0.00083 |
| Orange juice | -7.24E-02 | 0.007645 |  |
| Oranges |  | 0.122973 | -0.01278 |
| Pasta | -1.37E-02 | 0.031877 |  |
| Pears |  | 0.001643 | -0.00126 |
| Pizza |  | 0.023103 | -0.01258 |
| Plain yogurt | 5.70E-03 | -0.00789 |  |
| Plums | 3.38E-02 |  | -0.07278 |
| Pollock |  | 0.00698 | -0.02779 |
| Pork chops | -4.92E-02 | 0.012447 |  |
| Porridge | -1.65E-02 |  |  |
| Potatoes | -3.82E-02 |  | 0.062646 |
| Prawns |  | 0.034186 | -0.00499 |
| Raw carrots |  | -0.04659 | 0.001933 |
| Red meat | -2.63E-02 | 0.003498 |  |
| Red wine | 5.33E-03 |  | -0.01295 |
| Roast chicken | -4.24E-02 | 0.175508 |  |
| Salad dressing | | 0.021561 | -0.02145 |
| Salad leaves | 8.14E-02 | -0.02874 |  |
| Salami |  | 0.017745 | -0.00795 |
| Salmon |  | 0.02574 | -0.14787 |
| Salty foods | 1.77E-02 |  | -0.02445 |
| Salty pretzels | | 0.008328 | -0.00804 |
| Sardines | 7.92E-03 |  | -0.00248 |
| Sausages | -2.20E-02 | 0.007133 |  |
| Savoury biscuits | -7.48E-03 |  | 0.02533 |
| Shellfish | 5.09E-03 | -0.01725 |  |
| Skimmed milk | -1.19E-02 |  | 0.021531 |
| Smoked fish | 1.88E-05 |  | -0.00939 |
| Soft cheese |  | 0.015151 | -0.01471 |
| Soy milk | 2.57E-02 |  | -0.02292 |
| Soy sauce |  | 0.011915 | -0.00909 |
| Spicy foods | 2.36E-02 |  | -0.02889 |
| Spinach | 1.78E-01 |  | -0.00628 |
| Spirits |  | 0.010088 | -0.0056 |
| Strawberries | | 0.070987 | -0.30893 |
| Sweet coffee drinks | -1.27E-01 | 0.014672 |  |
| Sweet foods |  | 0.013377 | -0.00458 |
| Tea with sugar | ######## |  | 0.043778 |
| Tea without sugar | -4.36E-03 |  | 0.001594 |
| Tinned Tuna | -1.34E-02 | 0.017298 |  |
| Tomatoes | 9.29E-02 |  | -0.43413 |
| Turnip |  | -0.00669 | 0.004018 |
| Vegetables | 2.47E-01 | -0.11892 |  |
| Vinegar |  | -0.01726 | 0.015264 |
| Whisky | -2.35E-03 |  | 0.025113 |
| White bread | -3.05E-02 |  | 0.009825 |
| White rice | -3.61E-02 |  | 0.006462 |
| White wine |  |  | -0.02748 |
| Whole grain cereals | | 0.050938 | -0.02903 |
| Whole milk | 2.01E-03 | -0.00853 |  |
| Wholemeal bread | -6.33E-02 | 0.080688 |  |
