# Supplementary material for: Integrating Food Preference Profiling, Behavior Change Strategies, and Machine Learning for Cardiovascular Disease Prevention in a Personalized Nutrition Digital Health Intervention: Conceptual Pipeline Development and Proof-of-Principle Study
Source: J Med Internet Res. 2025 Aug 13;27:e75106. doi: 10.2196/75106 (PMC12346185; doi:10.2196/75106)
Supplement: Multimedia Appendix 3 [file jmir-v27-e75106-s003.docx]

Supplementary data 3. Feature important analysis using SHAP

| Feature | HC | | O | | ST | |
| --- | --- | --- | --- | --- | --- | --- |
|  | phi | phi.var | phi | phi.var | phi | phi.var |
| Add salt to food | 0 | 0 | 0 | 0 | 0 | 0 |
| Aniseed | 0 | 0 | 0 | 0 | 0 | 0 |
| Apple | 0 | 0 | 0 | 0 | 0 | 0 |
| Apple juice | 0 | 0 | 0 | 0 | 0 | 0 |
| Asparagus | 0 | 0 | 0.01 | 0.01 | -0.01 | 0.01 |
| Aubergine | -0.03 | 0.029394 | 0.01 | 0.01 | 0.02 | 0.019798 |
| Avocados | 0.01 | 0.01 | -0.01 | 0.01 | 0 | 0 |
| Bacon | 0.03 | 0.029394 | -0.03 | 0.049596 | 0 | 0.020202 |
| Baked/steam fish | 0 | 0 | 0 | 0 | 0 | 0 |
| Banana | 0 | 0 | 0 | 0 | 0 | 0 |
| BBQ grilled meat | 0.01 | 0.01 | 0.01 | 0.01 | -0.02 | 0.019798 |
| Beef steak | 0 | 0 | 0 | 0 | 0 | 0 |
| Beetroot | 0 | 0 | 0 | 0 | 0 | 0 |
| Bell pepper | 0.01 | 0.01 | 0 | 0 | -0.01 | 0.01 |
| Biscuits | 0 | 0 | -0.02 | 0.019798 | 0.02 | 0.019798 |
| Bitter ale | 0.01 | 0.01 | 0.01 | 0.01 | -0.02 | 0.019798 |
| Bitter foods | 0.01 | 0.01 | -0.01 | 0.01 | 0 | 0.020202 |
| Black olives | -0.01 | 0.01 | -0.01 | 0.01 | 0.02 | 0.019798 |
| Black pepper | 0 | 0 | 0 | 0 | 0 | 0 |
| Blue cheese | 0 | 0 | 0 | 0 | 0 | 0 |
| Bolognese sauce | 0 | 0 | 0 | 0 | 0 | 0 |
| Broad beans | 0 | 0 | 0 | 0 | 0 | 0 |
| Broccoli | 0 | 0 | 0.01 | 0.01 | -0.01 | 0.01 |
| Brown rice | 0 | 0 | 0 | 0 | 0 | 0 |
| Brussel sprout | 0 | 0 | 0.01 | 0.01 | -0.01 | 0.01 |
| Burgers | -0.01 | 0.01 | 0 | 0.020202 | 0.01 | 0.01 |
| Burn spicy | 0 | 0 | 0 | 0 | 0 | 0 |
| Butter on bread | 0 | 0 | 0 | 0 | 0 | 0 |
| Butternut squash | 0.01 | 0.01 | -0.01 | 0.01 | 0 | 0 |
| Cabbage | 0 | 0 | 0 | 0 | 0 | 0 |
| Cake | 0.01 | 0.01 | 0 | 0 | -0.01 | 0.01 |
| Cake icing | 0.01 | 0.01 | -0.01 | 0.01 | 0 | 0 |
| Capers | 0.01 | 0.01 | -0.01 | 0.01 | 0 | 0 |
| Cauliflower | 0 | 0 | 0 | 0.020202 | 0 | 0.020202 |
| Cereal bar | 0 | 0 | 0 | 0 | 0 | 0 |
| Cheesecake | 0 | 0 | 0 | 0 | 0 | 0 |
| Cherries | 0 | 0 | 0 | 0 | 0 | 0 |
| Chicken | 0 | 0.020202 | -0.04 | 0.038788 | 0.04 | 0.038788 |
| Chilli pepper | 0 | 0 | 0 | 0 | 0 | 0 |
| Chips | 0 | 0 | 0 | 0 | 0 | 0 |
| Cod | 0 | 0.020202 | -0.01 | 0.01 | 0.01 | 0.030202 |
| Coffee with sugar | -0.18 | 0.149091 | 0.06 | 0.05697 | 0.12 | 0.106667 |
| Coffee without sugar | 0 | 0 | -0.01 | 0.01 | 0.01 | 0.01 |
| Coriander | 0 | 0 | 0 | 0 | 0 | 0 |
| Cornflakes | -0.02 | 0.019798 | 0.02 | 0.019798 | 0 | 0.020202 |
| Cream | 0.02 | 0.019798 | -0.01 | 0.01 | -0.01 | 0.01 |
| Crisps | -0.01 | 0.01 | 0.01 | 0.01 | 0 | 0 |
| Croissant | 0 | 0 | -0.01 | 0.01 | 0.01 | 0.01 |
| Cucumber | -0.01 | 0.01 | -0.01 | 0.01 | 0.02 | 0.019798 |
| Curry | 0 | 0 | 0 | 0 | 0 | 0 |
| Dairy products | 0 | 0 | -0.01 | 0.01 | 0.01 | 0.01 |
| Darkchocolate | 0 | 0 | 0 | 0 | 0 | 0 |
| Diet fizzy drinks | -0.01 | 0.01 | 0 | 0 | 0.01 | 0.01 |
| Dried_fruit | 0 | 0.020202 | -0.01 | 0.01 | 0.01 | 0.01 |
| Eggs | 0 | 0 | 0 | 0 | 0 | 0 |
| EVO oil | 0 | 0 | 0 | 0 | 0 | 0 |
| Fatty foods | 0 | 0 | 0 | 0 | 0 | 0 |
| Fizzy drinks | 0 | 0 | 0 | 0 | 0 | 0 |
| Fried chicken | -0.02 | 0.04 | 0 | 0 | 0.02 | 0.04 |
| Fried fish | 0.01 | 0.030202 | -0.01 | 0.050404 | 0 | 0.020202 |
| Fruit | -0.05 | 0.04798 | -0.01 | 0.030202 | 0.06 | 0.05697 |
| Garlic | 0 | 0 | 0 | 0 | 0 | 0 |
| Gherkins | 0 | 0 | 0.01 | 0.01 | -0.01 | 0.01 |
| Globe artichoke | 0.01 | 0.01 | -0.01 | 0.01 | 0 | 0 |
| Goat cheese | 0 | 0 | 0 | 0 | 0 | 0 |
| Grapefruit | 0 | 0 | 0 | 0 | 0 | 0 |
| Green olives | 0 | 0 | 0 | 0 | 0 | 0 |
| Haddock | -0.01 | 0.01 | 0 | 0.020202 | 0.01 | 0.01 |
| Ham | -0.01 | 0.050404 | -0.03 | 0.029394 | 0.04 | 0.038788 |
| Hard cheese | 0 | 0 | 0 | 0 | 0 | 0 |
| Herring | 0 | 0 | 0 | 0 | 0 | 0 |
| Honey | 0 | 0 | 0 | 0 | 0 | 0 |
| Horseradish wasabi | 0 | 0 | -0.01 | 0.01 | 0.01 | 0.01 |
| Ice cream | 0 | 0 | 0 | 0 | 0 | 0 |
| Jam | 0 | 0 | 0 | 0 | 0 | 0 |
| Ketchup | 0 | 0 | 0 | 0 | 0 | 0 |
| Kiwi | 0 | 0 | 0.01 | 0.01 | -0.01 | 0.01 |
| Lager | 0 | 0 | 0.01 | 0.01 | -0.01 | 0.01 |
| Lamb | -0.01 | 0.01 | 0 | 0 | 0.01 | 0.01 |
| Lemons | 0 | 0 | -0.01 | 0.01 | 0.01 | 0.01 |
| Lentil beans | 0 | 0 | 0 | 0 | 0 | 0 |
| Liver | 0 | 0 | 0 | 0 | 0 | 0 |
| Mackerel | 0 | 0 | 0 | 0 | 0 | 0 |
| Marzipan | 0 | 0 | 0 | 0 | 0 | 0 |
| Mayonnaise | 0 | 0 | -0.01 | 0.01 | 0.01 | 0.01 |
| Melon | 0 | 0 | 0 | 0 | 0 | 0 |
| Milk chocolate | 0 | 0 | 0 | 0 | 0 | 0 |
| Mushroom | 0 | 0 | 0 | 0 | 0 | 0 |
| Onion | -0.01 | 0.01 | 0.01 | 0.01 | 0 | 0 |
| Orange juice | 0.01 | 0.01 | -0.02 | 0.019798 | 0.01 | 0.01 |
| Oranges | 0 | 0 | 0 | 0 | 0 | 0 |
| Pasta | 0 | 0 | 0 | 0 | 0 | 0 |
| Pears | 0 | 0 | 0 | 0 | 0 | 0 |
| Pizza | -0.01 | 0.01 | 0.01 | 0.01 | 0 | 0 |
| Plain yogurt | -0.02 | 0.04 | 0 | 0.020202 | 0.02 | 0.019798 |
| Plums | -0.02 | 0.019798 | 0.01 | 0.01 | 0.01 | 0.01 |
| Pollock | 0 | 0 | 0 | 0 | 0 | 0 |
| Pork chops | 0 | 0 | -0.01 | 0.01 | 0.01 | 0.01 |
| Porridge | 0 | 0 | 0 | 0 | 0 | 0 |
| Potatoes | -0.01 | 0.01 | 0 | 0 | 0.01 | 0.01 |
| Prawns | 0 | 0 | 0 | 0 | 0 | 0 |
| Raw carrots | 0 | 0 | 0 | 0 | 0 | 0 |
| Red meat | 0 | 0 | -0.01 | 0.01 | 0.01 | 0.01 |
| Red wine | 0 | 0 | 0 | 0 | 0 | 0 |
| Roast chicken | 0.03 | 0.029394 | -0.05 | 0.04798 | 0.02 | 0.019798 |
| Salad dressing | 0.01 | 0.01 | -0.01 | 0.030202 | 0 | 0.020202 |
| Salad leaves | -0.02 | 0.019798 | -0.01 | 0.01 | 0.03 | 0.029394 |
| Salami | -0.01 | 0.030202 | 0 | 0 | 0.01 | 0.030202 |
| Salmon | 0 | 0 | 0.01 | 0.01 | -0.01 | 0.01 |
| Salty foods | -0.01 | 0.01 | 0 | 0 | 0.01 | 0.01 |
| Salty pretzels | 0 | 0 | 0 | 0 | 0 | 0 |
| Sardines | 0 | 0 | 0.01 | 0.01 | -0.01 | 0.01 |
| Sausages | -0.01 | 0.01 | 0 | 0 | 0.01 | 0.01 |
| Savoury biscuits | 0 | 0 | 0 | 0 | 0 | 0 |
| Shellfish | 0.01 | 0.01 | 0 | 0 | -0.01 | 0.01 |
| Skimmed milk | 0 | 0 | 0 | 0 | 0 | 0 |
| Smoked fish | 0 | 0 | 0 | 0 | 0 | 0 |
| Soft cheese | 0 | 0.020202 | -0.01 | 0.01 | 0.01 | 0.01 |
| Soy milk | 0.01 | 0.01 | -0.01 | 0.01 | 0 | 0 |
| Soy sauce | 0 | 0 | 0 | 0 | 0 | 0 |
| Spicy foods | 0 | 0 | 0 | 0 | 0 | 0 |
| Spinach | -0.01 | 0.01 | 0 | 0 | 0.01 | 0.01 |
| Spirits | 0 | 0 | 0 | 0 | 0 | 0 |
| Strawberries | -0.08 | 0.074343 | -0.17 | 0.142525 | 0.25 | 0.189394 |
| Sweet coffee drinks | -0.05 | 0.04798 | 0.02 | 0.019798 | 0.03 | 0.029394 |
| Sweet foods | 0 | 0 | 0 | 0 | 0 | 0 |
| Tea with sugar | 0.03 | 0.029394 | 0.01 | 0.01 | -0.04 | 0.038788 |
| Tea without sugar | 0 | 0 | 0 | 0 | 0 | 0 |
| Tinned Tuna | 0 | 0 | 0 | 0 | 0 | 0 |
| Tomatoes | -0.01 | 0.01 | 0 | 0 | 0.01 | 0.01 |
| Turnip | 0 | 0 | 0 | 0 | 0 | 0 |
| Vegetables | -0.01 | 0.01 | 0.01 | 0.01 | 0 | 0 |
| Vinegar | 0 | 0 | 0.01 | 0.01 | -0.01 | 0.01 |
| Whisky | 0 | 0 | 0 | 0 | 0 | 0 |
| White_bread | -0.01 | 0.01 | 0 | 0 | 0.01 | 0.01 |
| White rice | -0.01 | 0.01 | 0 | 0.020202 | 0.01 | 0.01 |
| White wine | 0 | 0 | 0 | 0 | 0 | 0 |
| Whole grain cereals | 0.01 | 0.01 | 0 | 0 | -0.01 | 0.01 |
| Whole milk | 0 | 0 | 0 | 0 | 0 | 0 |
| Wholemeal bread | -0.01 | 0.01 | 0 | 0 | 0.01 | 0.01 |
