# Supplementary figures and images for: Integrating Food Preference Profiling, Behavior Change Strategies, and Machine Learning for Cardiovascular Disease Prevention in a Personalized Nutrition Digital Health Intervention: Conceptual Pipeline Development and Proof-of-Principle Study
Source: J Med Internet Res. 2025 Aug 13;27:e75106. doi: 10.2196/75106 (PMC12346185; doi:10.2196/75106)

Supplementary data 4. Feature important analysis using Random Forest


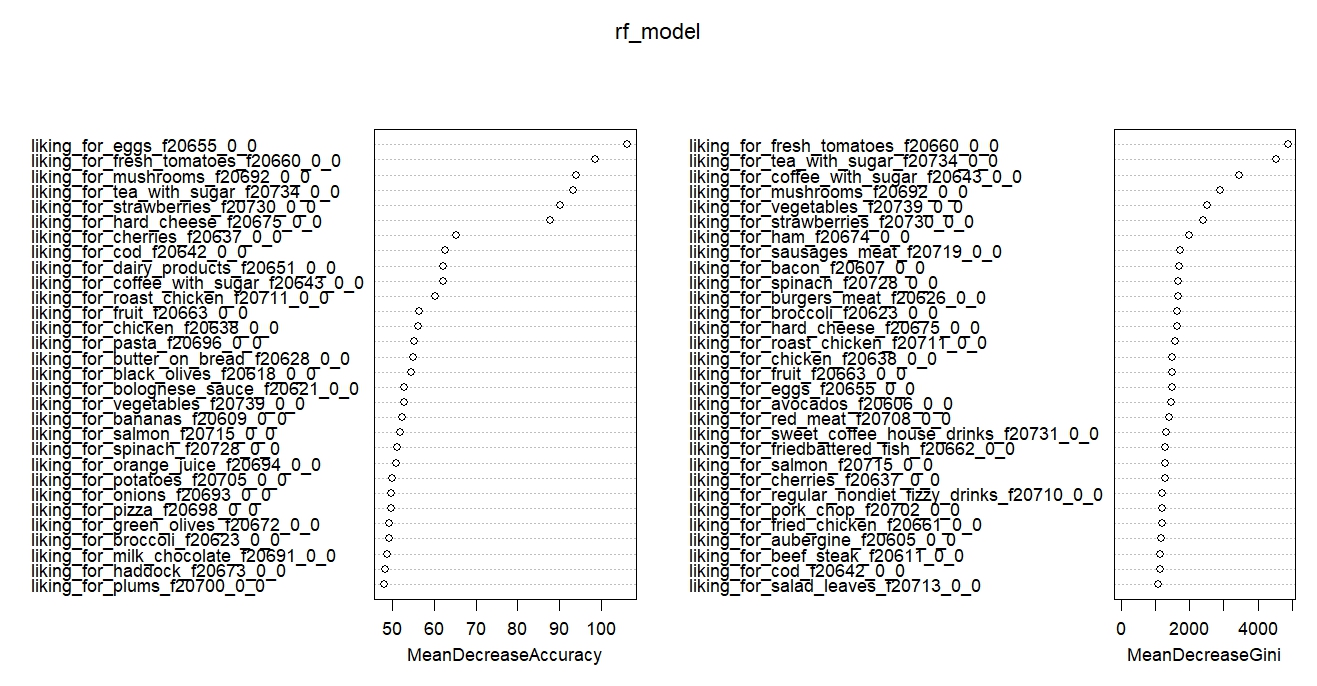

Supplement: Multimedia Appendix 4 [file jmir-v27-e75106-s004.docx]
