# Supplementary material for: Integrating Food Preference Profiling, Behavior Change Strategies, and Machine Learning for Cardiovascular Disease Prevention in a Personalized Nutrition Digital Health Intervention: Conceptual Pipeline Development and Proof-of-Principle Study
Source: J Med Internet Res. 2025 Aug 13;27:e75106. doi: 10.2196/75106 (PMC12346185; doi:10.2196/75106)
Supplement: Multimedia Appendix 5 [file jmir-v27-e75106-s005.docx]

Supplementary data 5. Comparison of CVD risk prediction performance using LDA between the full FPP model (140 food liking scores) and the simplified FPP model (14 food liking scores).

| Model | Accuracy | AUC | Precision-Recall |
| --- | --- | --- | --- |
| FPP_140 | 0.7513 | 0.7455 | 0.5745 |
| FPP_14 | 0.7513 | 0.7445 | 0.5737 |
